# Anwendung der Videosprechstunde in der kardiovaskulären Lipidbehandlung

**DOI:** 10.1007/s00059-023-05211-4

**Published:** 2023-10-19

**Authors:** A. Schuch, P. Walther, L. Timm, K. Steinbach, L. Haneklaus, T. Münzel, J. H. Prochaska, P. S. Wild

**Affiliations:** 1 Zentrum für Kardiologie, Kardiologie I, Universitätsmedizin der Johannes-Gutenberg-Universität Mainz, Mainz, Deutschland; 2 Deutsches Zentrum für Herz-Kreislauf-Forschung (DZHK), Standort Rhein-Main, 55131 Mainz, Deutschland; 3 Hochschule Fresenius, Hochschule für Angewandte Wissenschaften, 20095 Hamburg, Deutschland; 4 Forschungszentrum Translationale Vaskuläre Biologie (CTVB), Universitätsmedizin der Johannes-Gutenberg-Universität Mainz, Mainz, Deutschland; 5 Präventive Kardiologie und Medizinische Prävention, Zentrum für Kardiologie, Universitätsmedizin der Johannes-Gutenberg-Universität Mainz, Mainz, Deutschland; 6 Klinische Epidemiologie und Systemmedizin, Centrum für Thrombose und Hämostase, Universitätsmedizin der Johannes-Gutenberg-Universität Mainz, 55131 Mainz, Deutschland

**Keywords:** Lipidmanagement, Fernbehandlung, Arzt-Patienten-Kommunikation, Versorgungszugang, Informationsqualität, Lipid management, Remote treatment, Physician-patient communication, Care access, Information quality

## Abstract

**Hintergrund:**

Die Videosprechstunde ist eine Möglichkeit der ortsunabhängigen Arzt-Patienten-Kommunikation. Bei Möglichkeit der alleinigen Anwendung seit 2018 liegen jedoch nur eingeschränkte Informationen vor.

**Methoden:**

Nach Einführung der Videosprechstunde (Viomedi) in der Lipidsprechstunde der Universitätsmedizin Mainz wurden die Patienten im Q1-2022 nach Möglichkeit, Eignung und Bereitschaft zur Durchführung bewertet. Hierbei wurden Lipidpatienten und Long-COVID-Patienten eingeschlossen. Nach Behandlung wurde eine Onlinebefragung zu Nutzung und Einschätzung durchgeführt.

**Ergebnisse:**

29,1 % der 134 Patienten wurden eingeschlossen behandelt (3 Ablehnungen). Alle Probanden (16 Antworten) berichteten, (sehr) gut zurechtgekommen zu sein. Vorteile wurden in Beratung und Nachbeobachtung gesehen. Probleme wurden hinsichtlich Technik und möglicher Störungen befürchtet. Datenschutzaspekte spielten eine untergeordnete Rolle. Im Vergleich mit dem Telefonat wurde eine signifikante Verbesserung für das Arzt-Patienten-Verhältnis (*p*-Wert = 0,00027), die Behandlungs- und Informationsqualität (*p*-Wert jeweils = 0,00044), den Versorgungszugang (*p*-Wert = 0,0053) und die Kommunikation (*p*-Wert = 0,021) angenommen. Im Vergleich mit dem persönlichen Kontakt wurde eine Verbesserung des Versorgungszugangs (*p*-Wert = 0,021) und der Informationsqualität gesehen (*p*-Wert = 0,034). Als Hauptprobleme wurden fehlende Erfahrungen, technische Anforderungen, technische Probleme und fehlende Pünktlichkeit der Behandler angeführt. Gelobt wurden Flexibilität, geringer Aufwand und die angenehme Konsultation. Alle Probanden wollten die Videosprechstunde weiterhin nutzen.

**Fazit:**

Die Videosprechstunde kann eine Ergänzung der Versorgung von Lipidpatienten darstellen. Die korrekte Nutzung erfordert eine exakte Planung und weitere Forschung.

## Abstract (en)

**Background:**

Video consultation is a possibility for physician-patient communication independent of the location; however, only limited information is available for the possibility of sole use since 2018.

**Methods:**

After the implementation of video consultation (Viomedi) in lipid consultations at the Medical University Mainz, the patients in the first quarter of 2022 were assessed depending on the possibility, suitability and readiness to participate. Included were patients under lipid management and long COVID patients. After treatment an online survey was carried out on the utilization and appraisal.

**Results:**

Of the 134 patients 29.1% were inclusively treated (3 refusals). All subjects (16 replies) reported having managed (very) well. Advantages were seen in counselling and follow-up. Problems were feared with respect to technology and possible disorders. Data protection aspects played a subordinate role. In comparison to telephone calls, a significant improvement in the physician-patient relationship (*p*-value = 0.00027), the quality of treatment and information (*p*-value both = 0.00044), the access to care (*p*-value = 0.0053) and the communication (*p*-value = 0.021) was assumed. An improvement in access to care (*p*-value = 0.021) and the quality of information (*p*-value = 0.034) was seen in comparison to personal contact. The main problems were a lack of experience, technical requirements, technical problems and unpunctuality of the practitioner. The flexibility, low effort and the pleasant consultation were all praised. All subjects wanted to use the video consultation again.

**Conclusion:**

Video consultation can represent a supplement to treatment of patients under lipid management. The correct use requires exact planning and further research.

## Einleitung

Die Videosprechstunde ist eine Möglichkeit der ortsunabhängigen Arzt-Patienten-Kommunikation. Die Anwendung ist in Deutschland seit Änderung der Musterberufsordnung für die in Deutschland tätigen Ärztinnen und Ärzte (MBO) im Jahre 2018 möglich [34, S. 31]. Dieser rechtliche Rahmen macht eine Nutzung nach ärztlicher Beurteilung möglich. Eine unterstützende Anwendung ist grundsätzlich erlaubt. Eine ausschließliche Behandlung über diesen Weg ist nur in Einzelfällen erlaubt, wenn die Vorgehensweise vertretbar ist und die ärztliche Sorgfaltspflicht gewahrt bleibt (§ 7, Absatz 4, MBO).

Da erst vor Kurzem in Deutschland die Nutzung ermöglicht wurde, besteht nur eine geringe Erfahrungsgrundlage. Im Rahmen einer Forsa(Forsa Gesellschaft für Sozialforschung und statistische Analysen)-Umfrage konnte gezeigt werden, dass insbesondere Patienten den Wunsch nach einer ortsunabhängigen Kommunikation hegen. Dort äußerten 56 % der Patienten, dass sie sich die Nutzung der Videosprechstunde vorstellen könnten, 26 % wünschten sich eine dauerhafte Videosprechstunde. Auf Seiten der niedergelassenen Ärzte gaben lediglich 6 % der befragten Arztpraxen an, sich eine Anwendung vorstellen zu können [11, S. 46].

Seit Änderung der MBO hat sich die Nutzungsfrequenz deutlich erhöht. Der größte Anteil der aktiven Nutzer entfällt auf die Hausärzte [10, S. 1]. Durch die Coronapandemie konnten die Nutzungsfrequenz und die Akzeptanz weiter gesteigert werden [15, S. 632]. Jedoch ist die Videosprechstunde noch nicht fest in der ambulanten Versorgung etabliert [10, S. 1].

Auf Patientenseite bestehen Anforderungen an die Digitalkompetenz und die technische Ausstattung [20, S. 7]. Diese muss auch behandlerseitig sichergestellt werden, um eine sichere Anwendung zu ermöglichen [5, S. 15].

Das Patientenklientel der Kardiologie ist durch eine Gruppe mittleren bis höheren Alters hinsichtlich koronarer Herzkrankheit (KHK; [4, S. 2253]) und Herzinsuffizienz wie auch durch junge Erwachsene mit angeborenen Erkrankungen gekennzeichnet [27, S. 640]. Auch Fettstoffwechselstörungen können bereits bei jüngeren Patienten zu Problemen führen [31, S. 608]. Somit besteht ein heterogenes Patientenklientel. Für die Beurteilung der Anwendung der Videosprechstunde wird auf Basis einer Befragung das Meinungsbild der Patienten erhoben und ausgewertet.

## Methoden

In der Untersuchung wurden alle Patienten, die vom 01.01.2022 bis zum 31.03.2022 in der Lipidsprechstunde der Universitätsmedizin Mainz behandelt wurden, gescreent. Neben den Patienten der Lipidambulanz mit komplexer Hyperlipoproteinämie wurden seit Beginn 2022 auch Patienten mit Long-COVID(„coronavirus disease“)-Erkrankungen betreut. Somit lag eine gemischte Population vor.

Zunächst erfolgt eine ärztliche Einschätzung der Digitalkompetenz und der Eignung des Patienten sowie des Beratungsanlasses (§ 7, MBO). Die Digitalkompetenz wurde, basierend auf einer individuellen ärztlichen Einschätzung zur Kognition der Patienten, vorgenommen. Bei positiver Bewertung wurde dem Patienten eine Teilnahme offeriert. Es bestanden keine weiteren Ausschlusskriterien. Bei Nichtteilnahme wurden die Beweggründe erfragt. Die verbliebenen Patienten wurden via Videosprechstunde (Viomedi, Pilsting, Deutschland) untersucht. Es wurden die Vorgaben der Kassenärztlichen Bundesvereinigung (KBV) berücksichtigt [16, S. 2 ff.].

Für die Evaluation der Umsetzung der Videosprechstunde und der Patientenzufriedenheit wurde ein Fragebogen aus 20 Fragen mit 39 Items erstellt. Der Fragebogen findet sich im Supplement. Es handelt sich hierbei um einen Fragebogen aus geschlossenen, halboffenen und 2 offenen Fragen. Vereinzelt ist eine Mehrfachnennung möglich. Es werden demographische Daten, die körperliche Aktivität sowie die Internetnutzung erhoben. Es folgen Fragestellungen zur Qualität der Vorbereitung der Videosprechstunde und zu deren Durchführung. Im weiteren Verlauf des Fragebogens werden eine Patienteneinschätzung zu Risiko und Potenzial sowie eine Einordnung des Konsultationsweges im Vergleich zu einem persönlichen und einem telefonischen Kontakt festgehalten. Abschließend wird eine finale Bewertung erfragt und Raum für offenes Feedback angeboten. Die Bearbeitungsdauer des Fragebogens wurde auf 10–15 min taxiert. Für die Erhebung werden das Portal UmfrageOnline (https://www.umfrageonline.com/) sowie eine Word-basierte Formularversion verwendet. Die Probanden füllen den Fragebogen nach der Behandlung zu einem Zeitpunkt und an einem Ort ihrer Wahl aus.

Für die Analyse wurde Excel (Microsoft 365) und PSPP (PSPP_2020-09-05_daily_64bits) verwendet. Nicht vollständig ausgefüllte Fragebögen und solche, die nach dem 06.03.2022 eingegangen sind, wurden in der Analyse nicht berücksichtigt.

Die Auswertung erfolgte zunächst durch deskriptive Statistik und Häufigkeitsanalysen. Die Variablen wurden durch eine Mittelwertanalyse unter Annahme einer Normalverteilung analysiert. Neben der Analyse der einzelnen Items ist eine zusammengefasste Bewertung der Items unter Frage 5 in einer zu einem Mittelwert gruppierten Form vorgesehen.

Die Analyse erfolgt neben der Gesamtanalyse geschlechtsspezifisch und altersspezifisch (aufgeteilt in eine ältere und eine jüngere Gruppe) wie auch nach der Eigeneinschätzung zur körperlichen Aktivität in einer Gruppe, die sich als körperlich aktiver einschätzt, gegen eine Gruppe mit einer körperlich inaktiveren Einschätzung. Die körperliche Aktivität wird, ausgehend von Frage 5, analysiert. Die Alters- und Aktivitätsgruppen wurden orientiert am Median aller Antworten gebildet.

Die Patientensichten zu Risiken (Frage 14) und Potenzialen (Frage 15) wie auch eine vergleichende Analyse zu persönlichem Kontakt (Frage 16) und Telefonie (Frage 17) wurden unter Verwendung 2‑seitiger T‑Tests durchgeführt, jeweils pro Item und für die Frage als kumulierter, arithmetischer Mittelwert für die vergleichenden Analysen (Frage 16 und Frage 17). Die Analyse nach einem weiteren Nutzungswunsch erfolgte ausschließlich deskriptiv.

Von einer statistischen Signifikanz wird bei einem *p-*Wert unter 0,05 ausgegangen. Die Darstellung der Ergebnisse erfolgt unter Verwendung von Tabellen, Kuchen- und Balkendiagrammen sowie Boxplots, die durch die verwendeten Programme ausgegeben werden.

## Ergebnisse

**Figure 1:**
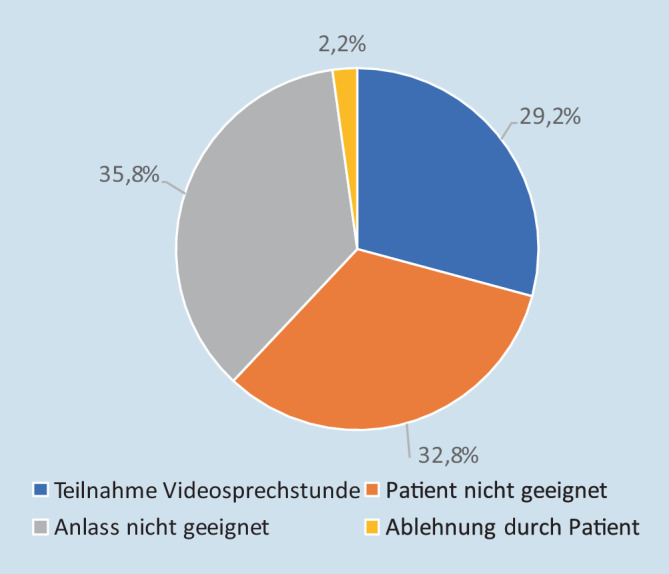

Von den 134 Patienten, die in Quartal (Q) 1/2022 behandelt wurden, partizipierten 39 Personen (29,1 %) an der Videosprechstunde. Hiervon handelte es sich gemäß systemseitiger Dokumentation bei 19 Patienten um Long-COVID-Patienten. Von den 95 Patienten, die nicht eingeschlossen wurden, waren bei 13 Patienten keine Arztkontakte erforderlich. Bei 35 Patienten waren Erstkontakte geplant, die eine körperliche Untersuchung erforderten. Von den verbleibenden 47 Patienten wurden 44 aufgrund unzureichender Digitalkompetenz oder eingeschränkter kognitiver Leistungsfähigkeit ausgeschlossen. Patientenseitig haben 3 Patienten unter Altersverweis abgelehnt. Die Patientenaufteilung zur Partizipation ist in Abb. 1 illustriert.

Von den eingeschlossenen Patienten beantworteten 16 Probanden (41 %) die Befragung. Das Alter lag im Mittel bei 51 ± 17,85 Jahren. Die Patienten waren zu 56,25 % männlich. Die weiblichen Patienten waren tendenziell jünger, was kein Signifikanzniveau erreichte. Männliche Probanden wie jüngere Probanden schätzten sich als aktiver ein, wobei kein Signifikanzniveau erreicht wurde. Eine Darstellung der Patientencharakteristika findet sich in Tab. 1.

**Table 1:** 

Variablen	Gesamte Population (*n* = 16)	Männlich (*n* = 9)	Weiblich (*n* = 7)	Ältere Probanden (*n* = 8)	Jüngere Probanden (*n* = 8)	Aktivere Patienten (*n* = 8)	Inaktivere Patienten (*n* = 8)
Alter in Jahren	51 ± 17,85	47,8 ± 16,63	53,44 ± 18,37	65,88 ± 10,93	36,13 ± 8,67	42,5 ± 12,89	59,5 ± 18,06
Männlich in Prozent (*n*)	56,25 % (9/16)	100 % (9/9)	0 % (0/7)	62,5 % (5/8)	50 % (4/8)	75 % (6/8)	37,5 % (3/8)
Eigenständigkeit im öffentlichen Raum in Prozent (*n*)	93,75 % (15/16)	88,89 % (8/9)	100 % (7/7)	87,5 % (7/8)	100 % (8/8)	100 % (8/8)	87,5 % (7/8)
Internetfähiges Handy	100 % (16/16)	100 % (9/9)	100 % (7/7)	100 % (8/8)	100 % (8/8)	100 % (8/8)	100 % (8/8)

Tabellendarstellung der Populationscharakteristika der befragten Probanden; bei kontinuierlichen Variablen erfolgt die Angabe einer Standardabweichung, bei dichotomen Variablen erfolgt die Angabe der Probandenanzahl (*n*). Die Darstellung erfolgt in der Gesamtpopulation sowie in den definierten Subgruppen

**Figure 2:**
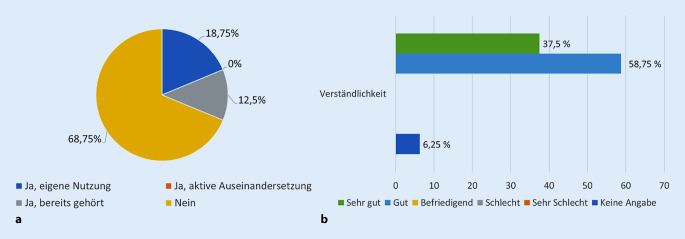

Von den Befragten gaben 31,25 % an, bereits von der Videosprechstunde erfahren zu haben. 18,75 % gaben an, bereits aktiv an einer Videosprechstunde teilgenommen zu haben. Bei der Befragung äußerten 93,75 % der Patienten, die Erklärungen „(sehr) gut“ verstanden zu haben (Abb. 2). Auch mit der folgenden Anwendung seien alle Patienten „(sehr) gut“ zurechtgekommen.

**Figure 3:**
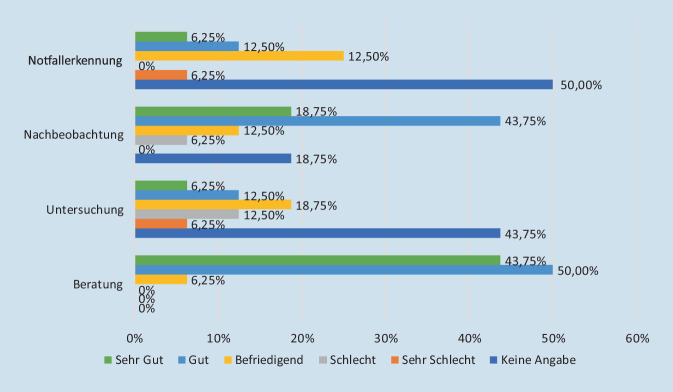

Die Patienten sahen die Vorteile in der Beratung und abgestuft in der Nachbeobachtung. Als weniger geeignet wurden Patientenuntersuchung und Notfallerkennung gewertet. Hier wurde insbesondere das Potenzial zur Notfallerkennung kritisch bewertet (Abb. 3).

**Figure 4:**
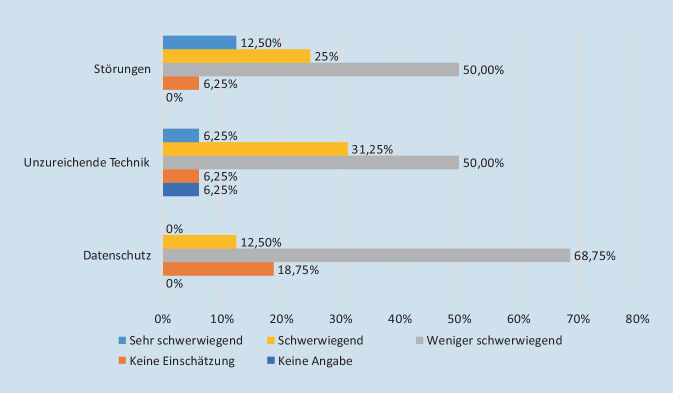

Probleme sahen die Befragten hinsichtlich Störungen und der technischen Ausstattung. Datenschutzaspekte spielten eine kleinere Rolle (Abb. 4). Eine statistische Signifikanz ist hierbei nicht nachweisbar.

**Figure 5:**
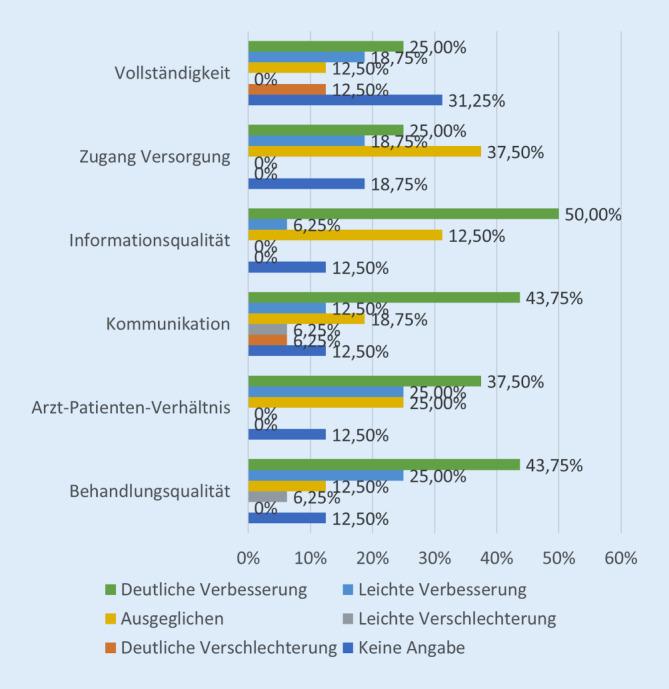

Die Probanden sahen eine Verbesserung gegenüber einem Telefonat in allen erhobenen Bereichen. Eine signifikante Verbesserung wurde für das Arzt-Patienten-Verhältnis (*p*-Wert = 0,00027), die Behandlungs- und Informationsqualität (*p*-Wert je = 0,00044) wie auch für den Versorgungszugang (*p*-Wert = 0,0053) und die Kommunikation (*p*-Wert = 0,021) berichtet (Abb. 5).

**Figure 6:**
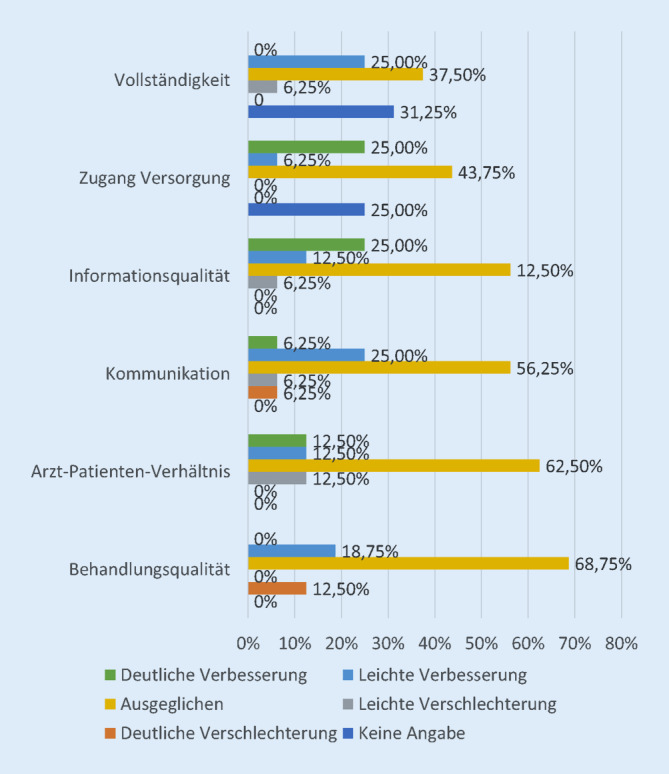

Weniger eindeutig wurde der Vergleich mit einem persönlichen Kontakt bewertet. Jedoch wurde auch hier eine Verbesserung des Versorgungszugangs (*p*-Wert = 0,021) und der Informationsqualität (*p*-Wert = 0,034) gesehen. Ein großer Anteil bewertete diesen Vergleich eher ausgeglichen. Kritisch wurden insbesondere die Kommunikation und das Arzt-Patienten-Verhältnis bewertet. Es fanden sich keine Hinweise auf eine signifikante Verschlechterung der Videosprechstunde gegenüber der Telefonie oder einem persönlichen Kontakt.

Bei Betrachtung der Subgruppen gab es leichte Abweichungen. So nahmen Männer häufiger an, dass die Videosprechstunde zur Vollständigkeit der Untersuchung beiträgt (*p*-Wert = 0,039). Im Vergleich mit dem telefonischen Kontakt schätzen männliche Probanden die Erleichterung des Versorgungszugangs höher ein (*p*-Wert = 0,047). Aktivere Probanden bewerteten die Verbesserung der Behandlungsqualität höher (*p*-Wert = 0,023).

Hervorgehoben wurden die zeitliche/örtliche Flexibilität, der geringe Aufwand für Patienten (keine Anreise, geringere Wartezeiten, vertrautes Umfeld), die unkomplizierte und verständliche Konsultation sowie das verringerte Infektionsrisiko (Möglichkeit eines Kontakts ohne Mund-Nasen-Schutz). Als problematisch führten die Patienten fehlende Erfahrung und Unsicherheiten sowie technische Anforderungen und technische Probleme der Universität an. Ein Proband beklagte ein zu kleines Display seines Mobiltelefons. Ferner wurde die fehlende Pünktlichkeit der Behandler kritisiert.

Zusammengefasst gaben alle Probanden an, eine weitere Nutzung zu wünschen.

## Diskussion

Die Bedeutung der Videosprechstunde hat während der COVID-19(„coronavirus disease 2019“)-Pandemie zugenommen. So wurde die Frage nach Möglichkeiten der Patientenversorgung mit minimalem direkten Kontakt aufgeworfen [3, S. 49]. Im Vordergrund steht neben der Anwendungsmöglichkeit die Patienteneignung, für die keine verbindlichen Kriterien definiert sind, wodurch eine Reproduzierbarkeit eingeschränkt ist. Zwar wurde bereits 2018 im Rahmen einer Novellierung der Berufsordnung das Fernbehandlungsverbot gekippt (§ 7, MBO). Unverändert sind jedoch Langzeitinformationen zu Effekten der Videosprechstunde insbesondere in der fachärztlichen Versorgung selten. Insbesondere in der kardiologischen Beratung und Notfallversorgung finden sich kaum belastbare Daten. Hinsichtlich der Beratungsanlässe wurden lediglich Elektivkontakte abgebildet, sodass keine Aussage zu Akutsituationen möglich ist. Durch die körperlichen Untersuchungen bei Erstkontakten konnten diese nicht virtuell angeboten werden.

Weiterhin erscheint eine Objektivierung der Behandlungsqualität diffizil, sodass diese Erhebung keine harten Endpunkte zur Bewertung bietet. Ein Problem der Nutzung besteht in technischen Anforderungen und notweniger Digitalkompetenz, die eine Nutzung für verschiedene Patientengruppen einschränken [19, S. 6]. Bei einer Befragung des Hartmannbundes sahen die Ärzte sowohl bei Patienten (56 %) als auch bei sich selbst (43 %) einen Nachholbedarf [40, S. 550].

Zuletzt wurde eine vermehrte Nutzung registriert. Neben der Pandemiesituation kann dies potenziell auf eine effizientere Patientenversorgung mit einem ortsunabhängigen, niederschwelligen Behandlungsangebot zurückgeführt werden [23, S. 832 f.]. Ein weiterer Faktor besteht in der Altersstruktur. Weiterhin ist in höherem Alter eine geringere Digitalkompetenz zu finden, jedoch ist auch in mittleren und höheren Altersgruppen eine steigende Sicherheit im Umgang mit digitalen Medien zu erkennen [1, S. 112 ff.], sodass ein Bedeutungsgewinn derartiger Angebote in der Patientenversorgung realistisch erscheint.

In der Kardiologie sind körperliche Untersuchungen und Diagnostika wie die Echokardiographie zentral. Andererseits ist in der Rhythmologie ein Telemonitoring in Anwendung [18, S. 171 ff.]. Bei der Lipidbehandlung spielen diese Aspekte lediglich eine untergeordnete Rolle. Die Lipidsprechstunde wurde somit als elektive Sprechstunde ohne die Notwendigkeit einer kardialen Bildgebung ausgewählt. Die Kardiologie selbst ist vielfältig, und daher ist eine Anwendung auch in anderen Sprechstunden vorstellbar [27, S. 640].

Initial wurde ausschließlich die Behandlung von Patienten mit einer komplexen Lipidtherapie intendiert. Im Zuge der Befragung wurden auch Long-COVID-Patienten in der Sprechstunde behandelt. Durch die Hinzunahme im Verlauf war eine Trennung nicht vorgesehen. Bei Long-COVID-Patienten scheint ein erhöhtes kardiales Risiko zu bestehen, sodass eine kardiologische Vorstellung zur Risikoevaluation diskutiert wird [9, S. 379]. Auch andere kardioembolische Ereignisse scheinen nach COVID-19-Erkrankung gehäuft vorzukommen [39, S. 583]. Es existieren Daten zu einem milderen Verlauf der Erkrankung unter Lipidtherapie [6, S. 145]. Eine finale Empfehlung steht aktuell aus. Dennoch ist bei Betrachtung von Long-COVID- und Lipidpatienten eine Verzerrung möglich.

Insgesamt wurden 39 Patienten behandelt, von denen 16 den Fragebogen ausfüllten. Zu den übrigen 23 Probanden ist keine Aussage möglich. Somit ist ein Selektionseffekt nicht auszuschließen. Wie sich im Rahmen der Analyse einer Befragung von Schulen zeigte, war bezüglich Gesundheitsthemen bei reduziertem Rücklauf kein Selektionseffekt erkennbar [22, S. 706]. Insbesondere Fragen, die eine Einschätzung erfordern, können eine Verzerrung bedingen [2, S. 10 f.]. Bei der Ergebnisinterpretation ist auch das multiple Testproblem relevant [12, S. 383 f.].

Es handelt sich um eine Erhebung in der Initialphase. Somit sind eine Unsicherheit und ein Lernprozess der Anwender möglich. Andererseits ist von einer höheren Aufmerksamkeit und einer detaillierteren Vorbereitung auszugehen [7, S. 323 f.]. Im Verlauf ist eine Veränderung der Werte möglich, sodass eine Reevaluation sinnvoll erscheint.

Die Probanden hatten hinsichtlich der Datensicherheit lediglich geringe Bedenken. Ein ähnliches Bild konnte in Vorbefragungen gefunden werden [29, S. 81]. Der Datenschutz ist bereits durch gesetzliche Grundlagen geregelt [8, S. 479], sodass der Stellenwert in der praktischen Anwendung eher von untergeordneter Bedeutung sein kann.

Ein hohes Potenzial sahen die Patienten in der Beratung und abgestuft in der Nachbeobachtung, da beide Thematiken weitgehend ohne taktile Komponente auskommen. Jedoch wurde bei der Nachbeobachtung von Tumoren eine höhere Abbruchrate festgestellt [36, S. 3 ff.], wohingegen bei der Betreuung von Depressions‑/Angstpatienten keine signifikanten Nachteile gefunden wurden [35, S. 1].

Konsultationen erfordern teilweise taktile Untersuchungen, sodass hier Einschränkungen möglich sind. Bei der Analyse von Handuntersuchungen konnte eine hohe Genauigkeit mit Einschränkungen bei kleinen Veränderungen und Läsionen gefunden werden [37, S. 2] Auch Wirbelsäulenuntersuchungen scheinen abbildbar zu sein. Jedoch sind Provokationstests anscheinend nicht sicher durchführbar [14, S. 199 f.]. Informationen zu kardiologischen Untersuchungen liegen nicht vor. Hierbei handelt es sich jedoch insbesondere um akustische und apparative Untersuchungen, bei denen eine Fernuntersuchung aktuell nicht sinnvoll durchführbar erscheint.

Neben Untersuchungen wurde auch die Notfallerkennung kritisch bewertet. Die Literatur liefert hier keine klare Aussage [21, S. 16]. Zentral sind die Erkennung eines Notfalls und die adäquate Behandlung [25, S. 443 ff.]. Aus dem fehlenden direkten Kontakt kann eine Verzögerung resultieren, sodass ein schlechteres Outcome möglich ist. Andererseits können durch einen engeren Kontakt eine höhere Sensitivität und eine frühere Reaktionsmöglichkeit entstehen. Somit ist eine weitere Evaluation erforderlich. In der Studie wurden keine Notfälle betrachtet.

Im Vergleich mit einem telefonischen Kontakt wurde eine deutliche Verbesserung der Beratungsqualität berichtet. Insbesondere die Qualität der Behandlung und der Information wie auch der Versorgungszugang und das Arzt-Patienten-Verhältnis wurden verbessert gesehen. Dieser Vorteil kann in der visuellen Information mit Verbesserung der Vertrauensbasis [32, S. 48]. Zudem können Informationen multimedial übertragen werden, wodurch die Verständlichkeit verbessert werden kann [28, S. 29 f.]. Durch einen ortsunabhängigen Kontakt, der keine Anreise des Patienten erfordert, kann zudem eine erleichterte Patientenbehandlung erreicht und der Versorgungszugang vereinfacht werden.

Im Vergleich mit dem persönlichen Kontakt fand sich in diesem Rahmen ein ausgeglichenes Bild. In der persönlichen Vorstellung existiert eine visuelle Ebene; hier fehlt der Videosprechstunde eine taktile Ebene [21, S. 14]. Ohne direkten Kontakt kann eine Konsultation potenziell unpersönlich wirken und eine Distanz zwischen Behandler und Patient erzeugen. Entsprechend wurden die Kommunikation und das Arzt-Patienten-Verhältnis kritischer bewertet als die vorbeschriebenen Kriterien, wobei hier keine Verschlechterung zum persönlichen Kontakt beschrieben wurde. Der Patient bewegt sich weiterhin in einem vertrauten Umfeld, sodass eine angenehmere Gesprächsatmosphäre in einer positiveren Wahrnehmung resultieren kann. So könnte auch der erlebte verbesserte Versorgungszugang erklärt werden. Weiterhin sahen die Probanden auch die Qualität der übermittelten Informationen verbessert. Dies kann potenziell aus einer verstärkten Nutzung einer Visualisierung der Untersuchungsergebnisse, über eine erleichterte Teilung des Bildschirms, resultieren. Auch ein erhöhtes Engagement bei einem neuen Prozess kann hierzu beigetragen haben [7, S. 323 f.].

In der Gesamtwertung der Einzelaspekte wurde die Qualität der Videosprechstunde auch gegenüber einem persönlichen Kontakt tendenziell besser bewertet, jedoch ohne Signifikanz, sodass hier keine finale Bewertung möglich erscheint.

In der geschlechtsspezifischen Analyse schnitt die Videosprechstunde bei Männern tendenziell besser ab als bei Frauen. Insgesamt waren die Bedenken bei Patientinnen ausgeprägter. Dies deckt sich mit Ergebnissen früherer Studien, denn eine größere Akzeptanz telemedizinischer Angebote bei männlichen Probanden konnte bereits in einer Analyse in Österreich festgestellt werden [24, S. 21]. Eine Erklärung auf Basis einer technischen Affinität gilt als umstritten [33, S. 49]. Hinsichtlich der Gesundheitskompetenz bestehen lediglich geringe Unterschiede zwischen Männern und Frauen, wobei sich eine leichte Tendenz zugunsten der weiblichen Bevölkerung herauslesen lässt, die jedoch kein Signifikanzniveau erreichte [26, S. 30].

Eine ähnliche Tendenz, jedoch ohne statistisch eindeutige Signifikanz, fand sich bei der altersspezifischen Analyse der Probanden. Hier schätzten die jüngeren Probanden die Möglichkeiten der Videosprechstunde höher ein, wohingegen bei den älteren Probanden die Risiken eine größere Rolle spielten. Auch hier konnten Voranalysen bereits ein ähnliches Bild erheben [24, S. 21]. Bekannt sind zudem eine geringere Nutzung digitaler Medien und eine reduzierte Digitalkompetenz, die bereits im Rahmen von Erhebungen dokumentiert werden konnten [1, S. 112 ff.].

Aspekte, die hier angeführt werden, betreffen Sicherheitsbedenken, den Aufwand und eine Komplexität, welcher sich ein Teil der Patienten, insbesondere betagte Personen, nicht mehr gewachsen [30, S. 4 f.]. Auch in der vorliegenden Untersuchung lehnten die Patienten, die nicht teilnehmen wollten, unter Verweis auf das eigene Alter ab.

Die größten Unterschiede in der aktuellen Untersuchung fanden sich im Vergleich der Subgruppen, die auf Basis der eigenen Einschätzung zur körperlichen Aktivität gebildet worden sind. Die Potenziale der Videosprechstunde wurden hierbei deutlich stärker von den Probanden hervorgehoben, die sich als aktiver sahen.

Allgemein wird die körperliche Aktivität als eine Möglichkeit zur Reaktion auf physiologische Alterungsprozesse beschrieben, wodurch auch die Kognition verbessert werden kann [38, S. 36], somit ist eine Korrelation zwischen Alter und körperlicher Inaktivität zu erwarten. Hinsichtlich der Effekte ist bei aktiveren Patienten von einem engeren Terminplan auszugehen, sodass durch den Wegfall von Reisezeiten und die gesteigerte Flexibilität eine bessere Integration von Arztterminen in den individuellen Alltag ermöglicht werden kann; in der Folge kann daraus eine positivere Wahrnehmung resultieren.

Ein grundlegendes Problem bei der Interpretation der Ergebnisse besteht in der Definition der Aktivitätsgruppen. Diese beruht auf einer untersuchungsspezifischen Selbsteinschätzung, sodass eine Übertragbarkeit auf andere Populationen erschwert erscheint. Es ist keine Aussage über eine tatsächliche Leistungsfähigkeit des Individuums möglich.

Ein Problem, welches auch die Patienten in der Befragung angaben, besteht in den technischen Anforderungen, die nicht von allen Patienten erfüllt werden können. Mit fortschreitender Digitalisierung ist eine Zunahme des Internetzugangs zu erkennen. Dieser Zusammenhang korreliert altersabhängig. Bei den Probanden des Altersspektrums der Lipidambulanz verfügen im Schnitt mehr als 90 % über einen Internetzugang [17, S. 9]. Aufgrund dessen ist eine Benachteiligung sozial schwächerer und älterer Patienten nicht unwahrscheinlich. In der aktuellen Anwendung konnten solche Probleme jedoch nicht beobachtet werden.

Ein nutzerunabhängiges Problem besteht in der Notwendigkeit einer stabilen Internetverbindung. In Deutschland können somit regionale Einschränkungen resultieren [13, S. 614]. Jedoch sind auch bei ausreichender Abdeckung Netzwerkprobleme möglich, deren Lösung essenziell für die Anwendung ist. Hinsichtlich der Bild- und Tonübertragung berichteten sowohl die Patienten als auch die Anwender eine hohe Übertragungsqualität, wie sie auch in den Zertifizierungskriterien der KBV gefordert ist [34, S. 31].

Nach Angaben der MitarbeiterInnen der Lipidambulanz wiesen die Patienten bei der Anwendung der Videosprechstunde einen höheren Anspruch an die Pünktlichkeit der Behandler auf. Dieser Eindruck ergab sich auch in der Patientenbefragung. Ein Grund für diese Tendenz kann in der Unsicherheit hinsichtlich der Anwendung und in der damit verbundenen Angst vor eigenen Fehlern bestehen. Diese Unsicherheit könnte sich somit als Unzufriedenheit dem Behandler gegenüber manifestieren.

Unabhängig von sonstigen Effekten, gaben alle Patienten an, die Videosprechstunde auch in Zukunft nutzen zu wollen. Eine Evaluation weiterer Anwendungsgebiete erscheint auf Basis der vorliegenden Informationen sinnvoll.

## Limitationen

Bei der Befragung ist einschränkend zu beachten, dass lediglich Patienten eines Spezialgebiets der Kardiologie behandelt wurden, wodurch eine Übertragung auf andere Behandlungsanlässe und Sprechstunden nicht ohne Weiteres möglich ist. Weiterhin wurden neben den Lipidpatienten auch Long-COVID-Patienten behandelt. Eine nachträgliche Trennung auf Grundlage der erfassten Daten ist nicht umsetzbar. Aktuell sind zudem keine harten Endpunkte erfasst worden, die eine Objektivierung der Ergebnisse zur Behandlungsqualität erlauben. Weiterhin ist die Stichprobe in der aktuellen Untersuchung als klein anzusehen, was die Aussagekraft einschränkt. Für eine optimierte Anwendung sind weitere Untersuchungen zu den Vorteilen der Videosprechstunde erforderlich.

## Fazit für die Praxis

- Die Videosprechstunde kann eine niederschwellige Ergänzung der fachärztlichen Versorgung bei Lipidpatienten darstellen, die aus Patientensicht eine Verbesserung der Behandlungsqualität ermöglichen kann.
- Die Patienten scheinen der technischen Neuerung grundsätzlich positiv gegenüberzustehen.
- Die optimale Nutzung erfordert jedoch eine exakte Planung und eine weitere Erforschung der Anwendung.

## References

[1] AG, Generali Deutschland (2017). Alltag und digitale Medien. Generali Altersstudie 2017.

[2] Aschemann-Pilshofer B, Premsberger E (2001). Wie erstelle ich einen Fragebogen. Leitf Prax.

[3] Barczok M (2021). Was tun mit Atemwegspatienten in Corona-Zeiten?. MMW Fortschr Med.

[4] Bönte M, von dem Knesebeck O, Siegrist J, Marceau L, Link C, McKinlay J (2007). Einfluss von Patientenalter und Patientengeschlecht auf ärztliche Entscheidungen bei koronarer Herzkrankheit. Dtsch Med Wochenschr.

[5] Butt U, Criée CP, Freitag A, Gappa M, Heimann T, Kardos P, Schöbel C, Stais P, Wilkens M, Worth H, Windisch W (2021). Gemeinsam die digitale Zukunft gestalten. Pneumologie.

[6] Daniels LB (2020) Statine gegen COVID-19-Infektionen. Dtsch Med Wochenschr 145:

[7] Euler D, Sloane PF (1998). Implementation als Problem der Modellversuchsforschung. Unterrichtswissenschaft.

[8] Frielitz FS, Storm N, Hiort O, Katalinic A, von Sengbusch S (2019). Die Erstellung eines Datenschutzkonzeptes: eine Anleitung für telemedizinische Versorgungsprojekte. Bundesgesundheitsblatt Gesundheitsforschung Gesundheitsschutz.

[9] Funke-Chambour M, Feldmeyer L, Hoepner R, Huynh-Do U, Maurer B, Rexhaj E, Geiser T (2021) Das Long-COVID-Syndrom – ein neues Krankheitsbild nach COVID-19-Infekt. Praxis 10.1024/1661-8157/a003678 34019446

[10] Gensorowsky D, Surmann B, Schmidt J, Greiner W (2021) Nutzungsgrad und Nutzergruppen der Online-Videosprechstunde in der ambulanten ärztlichen Versorgung – Eine Routinedatenanalyse. Gesundheitswesen 10.1055/a-1312-6439 33412593

[11] Gerlof H (2018). Videosprechstunde – Patienten wollen, Ärzte warten noch. Uro-News.

[12] Hommel G (1988). A stagewise rejective multiple test procedure based on a modified Bonferroni test. Biometrika.

[13] Ilgmann C, Störr A (2020). Telekommunikationsnetze in Deutschland – mit einem öffentlichen Unternehmen ausbauen. Wirtschaftsdienst.

[14] Jansen T, Gathen M, Touet A, Goost H, Wirtz DC, Burger C, Pflugmacher R, Welle K, Kabir K (2021). Spine examination during COVID-19 pandemic via video consultation. Z Orthop Unfall.

[15] Jorzig A (2020). Haftungsrisiken bei Telemedizin und Videosprechstunden. Gynäkologe.

[16] KBV (2021um) Videosprechstunde: telemedizinisch gestützte Betreuung von Patienten. https://www.kbv.de/html/videosprechstunde.php. Zugegriffen: 15. Aug. 2021

[17] Kortmann L, Hagen C, Endter C, Riesch J, Tesch-Römer C (2021). Internetnutzung von Menschen in der zweiten Lebenshälfte während der Corona-Pandemie: Soziale Ungleichheiten bleiben bestehen.

[18] Manninger-Wünscher M, Scherr D, Zirlik A (2021). Telemedizinische Applikationen in der Kardiologie. Kardiol Up2date.

[19] Mattos Sda S, Hazin SM, Regis CT et al (2015) A telemedicine network for remote paediatric cardiology services in north-east Brazil. Bull World Health Organ 93(12):881–7 10.2471/BLT.14.148874 PMC4669725 26668441

[20] Müller T (2020). Videosprechstunden sind nicht für alle die Lösung. Info Neurol Psychiatr.

[21] Nieser CC, Rippel A (2019). Würden telemedizinische Videosprechstunden (für Patienten, die sich subjektiv als Notfall ärztlich vorstellen) als Alternative zur persönlichen Arztkonsultation in Anspruch genommen?: Arzt- und Patientenbefragungen zu Videosprechstunden.

[22] Nübling M, Vomstein M, Haug A, Lindner A, Nolle I, Lincke HJ (2019). Rücklaufquote und Repräsentativität. Gibt es eine Selektion von Zufriedenen oder Unzufriedenen? Eine Analyse bei 3.000 Schulen. Gesundheitswesen.

[23] Pförringer D, Ansorg J, Osterhoff G, Dittrich F, Scherer J, de Jager U, Back DA (2020). Digitalisierung in Orthopädie und Unfallchirurgie: Stand 2020 in Klinik und Praxis. Unfallchirurg.

[24] Riedel M, Czypionka T, Kronemann F (2013). Telemedizin im österreichischen Gesundheitswesen.

[25] Riedl B, Peter W (2020). Hausarzt im Netz der Versorgung. Basiswissen Allgemeinmedizin.

[26] Schaeffer D, Berens EM, Gille S, Griese L, Klinger J, de Sombre S, Vogt D, Hurrelmann K (2021). Gesundheitskompetenz der Bevölkerung in Deutschland vor und während der Corona Pandemie: Ergebnisse des HLS-GER 2.

[27] Schmaltz AA, Bauer UMM (2013). Erwachsene mit angeborenen Herzfehlern. Herz.

[28] Schmohl T (2019). Selbstgesteuertes Lernen. Explorative hochschuldidaktische Formate mit Modellcharakter für vier akademische Statusgruppen.

[29] Schnack D (2020). Die Videosprechstunde kommt an. Fokus Onkol.

[30] Seifert A (2021). Digitale Transformation in den Haushalten älterer Menschen. Z Gerontol Geriat.

[31] Sinning D, Landmesser U (2017). Fettstoffwechselstörungen. Herz.

[32] Sonnet M (2020). Diabetestherapie per Videosprechstunde. Info Diabetol.

[33] Stemmann J (2019). Gendergerechte Technik – eine Herausforderung für das Lernen in einer digitalen Welt?. J Tech Educ.

[34] Tenbrock R (2020). Videosprechstunden – Fluch oder Segen?. Orthop Rheumatol.

[35] Tönnies J, Hartmann M, Wensing M, Szecsenyi J, Peters-Klimm F, Brinster R, Weber D, Vomhof M, Icks A, Friederich HC, Haun MW (2021). Mental health specialist video consultations versus treatment-as-usual for patients with depression or anxiety disorders in primary care: randomized controlled feasibility trial. JMIR Ment Health.

[36] Walle T, Erdal E, Mühlsteffen L, Singh HM, Gnutzmann E, Grün B, Hofmann H, Ivanova A, Köhler BC, Korell F, Mavratzas A, Mock A, Pixberg C, Schult D, Starke H, Steinebrunner N, Woydack L, Schneeweiss A, Dietrich M, Jäger D, Krisam J, Kather JN, Winkler EC (2020). Completion rate and impact on physician-patient relationship of video consultations in medical oncology: a&nbsp;randomised controlled open-label trial. ESMO Open.

[37] Welle K, Täger S, Hackenberg RK, Markowetz A, Schildberg FA, Burger C, Wirtz DC, Jansen T, Kabir K (2021). Examining the Hand in the Video Consultation. Z Orthop Unfall.

[38] Wöhl C, Siebert H, Blättner B (2018). Körperliche Aktivität zur Stärkung kognitiver Ressourcen. Präv Gesundheitsf.

[39] Xie Y, Xu E, Bowe B, Al-Aly Z (2022). Long-term cardiovascular outcomes of COVID-19. Nat Med.

[40] Zielke R (2021). Krisengewinner Digitalisierung. Urologe.

